# Aerodynamic Instability Mechanisms of Iced Eight-Bundled Conductors: Frequency-Domain Analysis and Stability Assessment via Wind Tunnel–CFD Synergy

**DOI:** 10.3390/s25134120

**Published:** 2025-07-01

**Authors:** Bolin Zhong, Minghao Qiao, Mengqi Cai, Maoming Hu

**Affiliations:** 1 School of Architecture and Civil Engineering, Chengdu University, Chengdu 610106, China; 2 School of Mechanical Engineering, Chengdu University, Chengdu 610106, China

**Keywords:** iced conductors, aerodynamic characteristics, Fourier fitting, harmonic order analysis

## Abstract

Icing on transmission lines in cold regions can cause asymmetry in the conductor cross-section. This asymmetry can lead to low-frequency, large-amplitude oscillations, posing a serious threat to the stability and safety of power transmission systems. In this study, the aerodynamic characteristics of crescent-shaped and sector-shaped iced eight-bundled conductors were systematically investigated over an angle of attack range from 0° to 180°. A combined approach involving wind tunnel tests and high-precision computational fluid dynamics (CFD) simulations was adopted. In the wind tunnel tests, static aerodynamic coefficients and dynamic time series data were obtained using a high-precision aerodynamic balance and a turbulence grid. In the CFD simulations, transient flow structures and vortex shedding mechanisms were analyzed based on the Reynolds-averaged Navier–Stokes (RANS) equations with the SST *k*-*ω* turbulence model. A comprehensive comparison between the two ice accretion geometries was conducted. The results revealed distinct aerodynamic instability mechanisms and frequency-domain characteristics. The analysis was supported by Fourier’s fourth-order harmonic decomposition and energy spectrum analysis. It was found that crescent-shaped ice, due to its streamlined leading edge, induced a dominant single vortex shedding. In this case, the first-order harmonic accounted for 67.7% of the total energy. In contrast, the prismatic shape of sector-shaped ice caused migration of the separation point and introduced broadband energy input. Stability thresholds were determined using the Den Hartog criterion. Sector-shaped iced conductors exhibited significant negative aerodynamic damping under ten distinct operating conditions. Compared to the crescent-shaped case, the instability risk range increased by 60%. The strong agreement between simulation and experimental results validated the reliability of the numerical approach. This study establishes a multiscale analytical framework for understanding galloping mechanisms of iced conductors. It also identifies early warning indicators in the frequency domain and provides essential guidance for the design of more effective anti-galloping control strategies in resilient power transmission systems.

## 1. Introduction

Overhead transmission lines in cold and humid regions are frequently subjected to atmospheric icing, which alters their geometric profiles and introduces complex aerodynamic responses. Ice accretion typically transforms the conductor cross-section from a circular shape to asymmetric profiles, such as crescent, D-type, or sector-shaped configurations. These geometric changes significantly affect the aerodynamic forces acting on the conductors and can induce low-frequency, large-amplitude self-excited oscillations, commonly referred to as galloping. Galloping poses serious risks to the mechanical integrity of conductors and insulators, and it can compromise the safe operation of transmission systems. Under extreme conditions, it may lead to flashovers, damage to spacers, or even tower collapse [1,2,3].

### 1.1. Experimental Investigations

Wind tunnel tests employing high-frequency force balances have been a primary method for evaluating the aerodynamic behavior of iced conductors. These experiments aim to quantify the influence of key parameters, including strand geometry, ice accretion thickness, and ambient turbulence intensity, on the resulting aerodynamic coefficients [1,2,3]. The empirical data obtained from such tests are essential for understanding the complex aerodynamic forces acting on conductors under icing conditions. Moreover, they provide critical benchmarks for validating computational models.

### 1.2. Computational Fluid Dynamics—CFD Advancements

Computational fluid dynamics (CFD) methods, particularly those based on Reynolds-averaged Navier–Stokes (RANS) and unsteady Reynolds-averaged Navier–Stokes (URANS) formulations, have been widely employed to simulate the complex flow fields around iced conductors [4,5,6,7]. To improve the accuracy of these simulations, especially in capturing dynamic responses, advanced techniques have been increasingly adopted. These include the application of overset mesh strategies to accommodate complex moving geometries, and the integration of fluid–structure interaction (FSI) models to represent the coupled dynamics between the airflow and conductor motion [8,9].

### 1.3. Multimodal Analysis and Complex Dynamics

Traditional quasi-steady approaches, such as the Den Hartog criterion, are limited in their ability to capture the inherently unsteady and nonlinear aerodynamic behavior of iced conductors. These methods often lead to underestimations, particularly due to factors such as icing shape, surface roughness, turbulence, and flow separation [10,11,12,13]. To address these limitations, recent research has shifted toward more advanced analytical frameworks. A notable development involves the integration of time-domain simulation techniques with advanced modal decomposition methods [14,15,16,17]. This combined approach enables a more comprehensive investigation of complex phenomena, including multi-mode coupling during galloping and the detailed evolution of conductor trajectories.

### 1.4. Research Significance and Methods

Despite recent advances, a unified framework that integrates high-fidelity simulations with experimental validation—particularly for unsteady aerodynamic force characterization—remains an active area of research. Systematic investigations covering the full spectrum of aerodynamic responses across critical wind attack angles are still limited, especially for complex asymmetric ice geometries such as the sector shape [18,19,20]. Detailed analyses of unsteady aerodynamic forces and their connection to underlying flow physics are also lacking. To address these gaps, a hybrid approach is adopted in this study. Full-scale (1:1) wind tunnel tests are conducted in conjunction with CFD simulations to evaluate the aerodynamic performance of iced conductors. The wind tunnel tests employ a precision force measurement system with adjustable angle-of-attack control, enabling measurements across a wide range of angles relevant to galloping assessment. The numerical simulations are performed using a RANS-based solver with the SST *k*-*ω* turbulence model and structured meshes verified through a mesh independence study. To investigate the dynamic characteristics of aerodynamic loading, Fourier decomposition is applied to both experimental and numerical data. This allows extraction of dominant harmonic components and provides insight into the periodicity and energy distribution of unsteady forces, which are often associated with galloping excitation. Furthermore, Den Hartog coefficients are computed across the full range of wind attack angles to evaluate the potential for negative aerodynamic damping. These findings are then correlated with pressure field distributions and flow structure visualizations to enhance the understanding of galloping onset mechanisms.

## 2. Materials and Methods

### 2.1. Wind Tunnel Test Design

**Figure 1:**
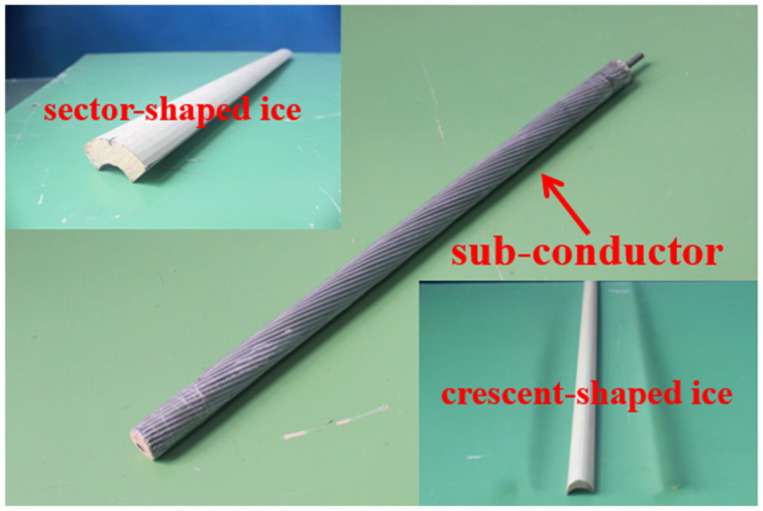
A steel-core aluminum stranded conductor and ice model.

**Figure 2:**
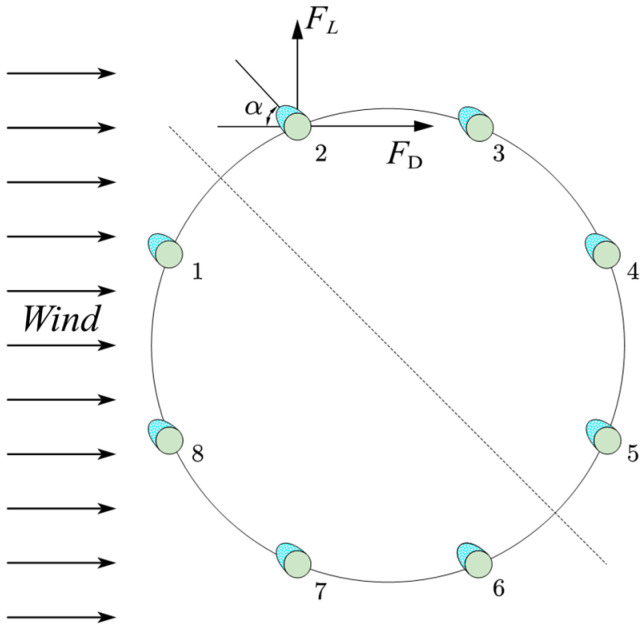
Layout of conductors. (The green parts represent the conductor, and the blue parts represent the ice. The numbers 1–8 represent the conductor numbers.)

In this study, aerodynamic tests were conducted in a low-speed wind tunnel using a full-scale conductor model (1:1 scale). The use of a full-scale model eliminated scaling effects, ensuring that the results are directly applicable to real transmission lines. As shown in Figure 1, the test model consisted of LGJ500/35 steel-core aluminum stranded conductors, with a diameter of 30 mm and a length of 1 m. This conductor type is widely used in practice, and the 1 m length was selected to minimize end effects and facilitate accurate two-dimensional flow measurements. Two typical ice accretion profiles were examined: a crescent shape (ice thickness = 12 mm) and a sector shape (ice thickness = 18 mm). These profiles were selected based on field observations and previous studies due to their known propensity to induce galloping. Eight conductors were arranged in an octagonal configuration with a 400 mm spacing, as illustrated in Figure 2. This spacing reflects common industry standards for bundled conductor arrangements.

**Figure 3:**
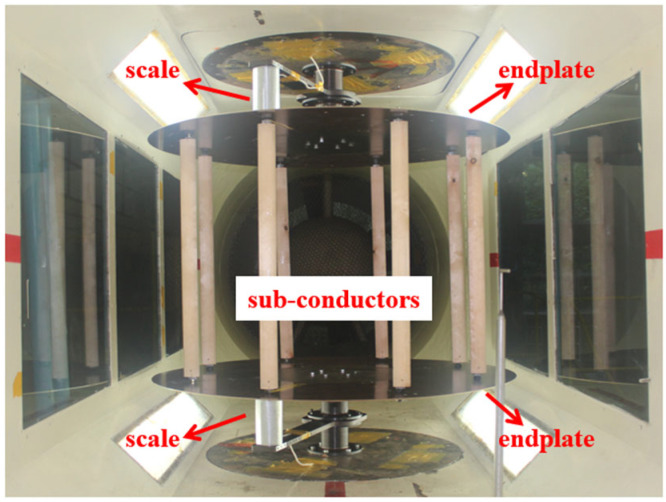
Wind tunnel test arrangement.

The experimental setup consisted of a high-precision DXTP sextuple-force pneumatic balance, a turbulence-generating grid, and a conductor rotation adjustment mechanism. To simulate the turbulent conditions typically encountered in actual operating environments, a grid-type turbulence generator was installed upstream of the test section, maintaining the incoming turbulence intensity at approximately 5%. The conductor model was mounted vertically at the center of a circular end plate, which was rigidly connected to the upper and lower turntables of the wind tunnel. The DXTP balance was affixed to this supporting structure through connecting plates and linked to both ends of the conductor model. A windshield was placed outside the balance to minimize external disturbances. All aerodynamic responses were recorded using a multi-channel signal acquisition system. The angle of attack was varied from 0° to 180° in 10° increments, resulting in 19 test conditions. For each angle, 10 s of data were collected at a constant wind speed of 10 m/s, with a sampling frequency of 1 kHz. The 10 s duration was carefully selected to ensure the capture of several complete cycles of low-frequency aerodynamic force fluctuations, which are critical for galloping analysis and typically occur below 100 Hz. For all angles tested, the data acquisition period was confirmed to include multiple stable vortex-shedding cycles or quasi-periodic responses, thus providing statistically representative data for both time-averaged and dynamic aerodynamic characteristics, including Den Hartog coefficients. A wind speed of 10 m/s was selected as the primary test condition due to its relevance to galloping-prone scenarios and its compatibility with both experimental and numerical analyses. Previous studies have shown that conductor galloping frequently initiates at moderate wind speeds (5–15 m/s), where aerodynamic damping transitions from positive to negative, making this range critical for evaluating ice-induced galloping behavior [21,22]. Testing at 10 m/s allowed for effective characterization of this instability-prone regime while avoiding the complications of high turbulence at higher speeds. All raw measurement data were subjected to low-pass filtering prior to post-processing to determine both average and instantaneous aerodynamic characteristics. The complete wind tunnel arrangement is illustrated in Figure 3.

### 2.2. Numerical Simulation Framework

#### 2.2.1. Parameter Setting

**Figure 4:**
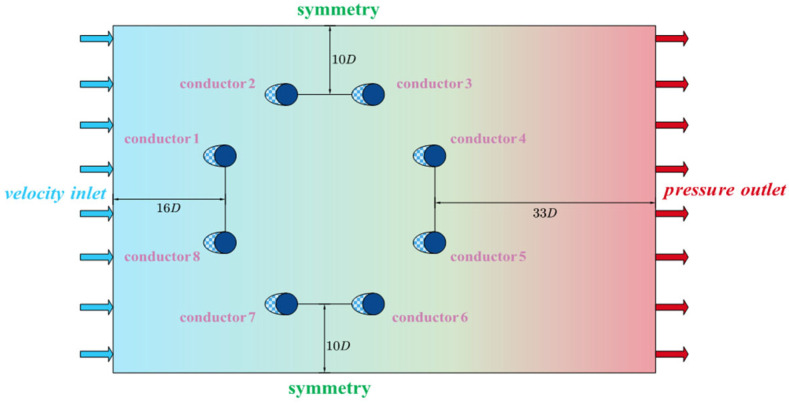
Flow field domain.

CFD simulations were performed using ANSYS Fluent 2023 R1. The governing equations were the RANS equations, with turbulence modeled using the SST *k*-*ω* model. This model offers a favorable balance between near-wall accuracy and flow separation prediction capabilities [23]. Steady RANS with the SST *k*-*ω* model was selected, as it reproduced the mean aerodynamic coefficients and dominant low-frequency harmonics within 10% of the experimental results, while requiring significantly less computational time and memory—approximately one order of magnitude less—compared to URANS or LES approaches. This provided an effective trade-off between accuracy and computational cost for simulations across the full 0–180° angle of attack range. The computational domain was designed to minimize boundary effects and ensure fully developed flow conditions. The conductor model was positioned 16 D (where D is the conductor diameter) from the inlet, 33 D from the outlet, and 10 D from both the upper and lower domain boundaries. These dimensions, as illustrated in Figure 4, were carefully selected to allow for proper flow development upstream of the model and sufficient wake dissipation downstream, thus avoiding artificial interference. Boundary conditions were set as follows: the inlet was defined as a velocity inlet to impose a uniform incoming flow; the outlet was specified as a pressure outlet to simulate an open boundary; the upper and lower boundaries were treated as sliding walls to emulate an infinite vertical domain and minimize wall-induced effects. A time step of 0.00025 s was employed. This value was determined through sensitivity analysis to ensure computational stability while capturing the unsteady aerodynamic phenomena with adequate temporal resolution. It was chosen to resolve the smallest relevant turbulent structures and the dominant vortex shedding frequencies associated with dynamic pressure variations around the iced conductors.

#### 2.2.2. Mesh Delineation and Validation

A structured mesh was employed for the entire computational domain. An “O”-type mesh topology was applied around each conductor to enhance near-wall resolution and improve the accuracy of boundary layer predictions. To assess mesh independence, three mesh densities—2 million, 4 million, and 6 million cells—were tested. The criterion for mesh convergence was that the variation in key aerodynamic coefficients, specifically the lift coefficient of conductor 1, remained within 3%. Based on this evaluation, the 4 million-cell mesh was selected for both the crescent-shaped and sector-shaped iced eight-bundled conductor models, as it offered a favorable balance between accuracy and computational efficiency. The results of the mesh independence study are summarized in Table 1.

## 3. Methods of Data Analysis

A multi-level, multi-angle data processing framework was adopted to comprehensively evaluate the aerodynamic responses of iced conductors and to assess their susceptibility to galloping. The analysis included the extraction of time-averaged aerodynamic coefficients, the characterization of unsteady aerodynamic behavior, and the quantification of galloping stability parameters [24,25]. These combined methods ensured the accuracy and reliability of the results.

### 3.1. Calculation of Average Lift/Drag Coefficient

The aerodynamic forces acting on the conductors—lift and drag—are represented by the lift coefficient (*C_l_*) and drag coefficient (*C_d_*), respectively [26,27], and are defined as follows:

(1) $$C_{l}=\frac{F_{l}}{0.5\rho U^{2}DL}$$

(2) $$C_{d}=\frac{F_{d}}{0.5\rho U^{2}DL}$$

Here, *F_l_* and *F_d_* denote the instantaneous lift and drag forces, respectively. *ρ* represents the air density, taken as 1.225 kg/m^3^ under standard atmospheric conditions. *U* is the free-stream velocity, maintained at a constant 10 m/s. *D* refers to the equivalent diameter of the conductors, including the ice accretion—42 mm for the crescent-shaped profile and 48 mm for the sector-shaped profile. *L* denotes the conductor length, which is uniformly set to 1 m. Steady-state aerodynamic coefficients at each angle of attack were obtained by time-averaging the measured, time-resolved aerodynamic signals. This process provides the foundation for subsequent analyses of unsteady behavior and stability characteristics. By plotting the lift and drag coefficients against the angle of attack, key aerodynamic features can be identified, including the onset of flow turning, the critical stall angle, and the maximum lift point. These trends reveal the influence of different ice accretion profiles on the overall aerodynamic performance of the conductors [28,29,30].

### 3.2. Aerodynamic Dynamic Characterization

In wind-induced vibration studies, the aerodynamic forces acting on conductors often exhibit pronounced non-stationary characteristics. Periodic variations in vortex shedding and wake structures generate strong oscillations in both lift and drag forces. To accurately capture these periodic features and identify dominant frequency components, the Fourier series expansion method is employed. Time-resolved aerodynamic signals at each angle of attack are analyzed in the frequency domain. Based on the results, an aerodynamic model is constructed using the first through fourth-order harmonic components:

(3) $$F\left(t\right)\approx a_{0}+\overset{4}{\underset{n=1}{\sum }}\left[a_{n}\cos\left(n\omega t\right)+b_{n}\sin\left(n\omega t\right)\right]$$

Here, $\;F\left(t\right)\;$ denotes the instantaneous aerodynamic force on one side of the conductor. $\;a_{0}\;$ represents the DC component of the aerodynamic signal, i.e., the time-averaged value. $\;a_{n}\;$ and $\;b_{n}\;$ denote the amplitude coefficients of the nth-order cosine and sinusoidal terms, respectively. *ω* is the dominant angular frequency,   t  denotes the time, and n  denotes the harmonic order. In this study, *n* = 1 to 4 is selected to capture the primary periodic components of the aerodynamic signal. The choice to include harmonics up to the fourth order is based on a comprehensive analysis of the signal’s energy distribution. These initial harmonics were found to account for the vast majority of the total energy in the aerodynamic force signal. As such, they provide a sufficient representation of the dominant oscillatory behavior, while avoiding unnecessary complexity from higher-order terms that contribute minimally. This approach is consistent with established practices in aerodynamic force decomposition, where emphasis is placed on identifying and quantifying the most energetic and influential frequency components relevant to stability analysis. The amplitudes of each harmonic were compared to extract the dominant frequency and evaluate the corresponding energy distribution. This analysis clarified the effect of angle of attack on both periodic and non-stationary aerodynamic force characteristics. In addition, low-frequency and subharmonic components were identified, indicating potential resonance risks and shedding light on underlying flow separation mechanisms.

### 3.3. Anti-Galloping Stability Assessment

To quantify the anti-galloping performance of iced conductors under incoming wind flow, the Den Hartog stability criterion was employed to evaluate the aerodynamic behavior at each angle of attack [31,32,33]. The Den Hartog coefficient is defined as follows:

(4) $$H=\frac{dC_{l0}}{d\alpha }+C_{d0}$$

Here, *H* denotes the Den Hartog coefficient and α represents the angle of attack. $\;C_{l0}\;$ and $\;C_{d0}\;$ refer to the lift and drag coefficients, respectively, at a given angle of attack. A negative value of *H* (*H* < 0) indicates the potential for negative aerodynamic damping, suggesting that the conductor is susceptible to galloping under that specific flow condition. By combining experimental and numerical data, the variation of *H* with respect to the angle of attack was obtained through numerical differentiation. The resulting *H*-*α* curve was used to identify critical regions where galloping is likely to occur, thereby providing a quantitative basis for assessing aerodynamic instability.

## 4. Results and Discussion

### 4.1. Static Aerodynamic Characteristics

**Figure 5:**
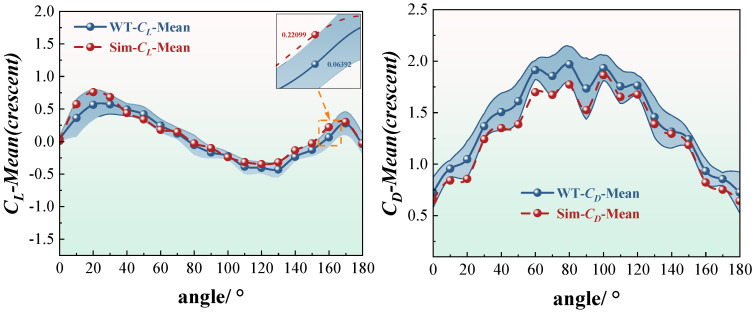
Average lift coefficient and drag coefficient of crescent-shaped iced eight-bundled conductors. (The blue striped area represents the result range for conductors 1–8 in the wind tunnel test.)

**Figure 6:**
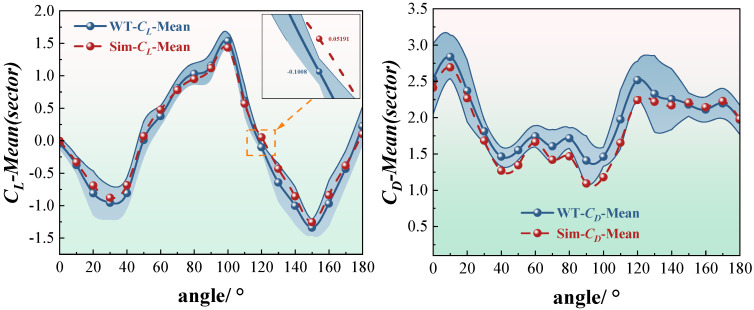
Average lift coefficient and drag coefficient of sector-shaped iced eight-bundled conductors. (The blue striped area represents the result range for conductors 1–8 in the wind tunnel test.)

To investigate the static aerodynamic response of iced conductors at varying angles of attack, this study compares the distribution patterns of lift and drag coefficients for two typical ice accretion profiles: crescent-shaped and sector-shaped. The average aerodynamic coefficients obtained from wind tunnel tests (WT) and numerical simulations (Sim) over the angle-of-attack range from 0° to 180° are presented in Figure 5 and Figure 6. The results demonstrate good agreement between the experimental and simulated data across most angles of attack, thereby confirming the reliability of the numerical approach in reproducing the static aerodynamic behavior of iced conductors.

For the crescent-shaped iced conductors (Figure 5), the lift coefficient exhibits a distinctly asymmetric distribution. The maximum lift occurs near an angle of attack of approximately 20°, with a peak value of *C_l_* ≈ 1.3. As the angle increases, the lift coefficient gradually decreases, approaching zero near 90°, and reaching negative peaks between 120° and 150°. This forms a characteristic asymmetric bimodal pattern, indicating a significant difference in aerodynamic force distribution between windward and leeward conditions for this ice accretion geometry. In contrast, the drag coefficient displays a nearly symmetric distribution. It increases gradually with the angle of attack, peaking between 80° and 100°, with a maximum value of approximately *C_d_* ≈ 2.1, followed by a slight decline at higher angles. The numerical simulation results closely replicate the experimental trends near both lift and drag peaks. The maximum relative error remains within 5%, demonstrating that the SST *k*-*ω* turbulence model performs robustly in capturing flow separation and stall characteristics associated with irregular ice-covered conductor surfaces.

For the sector-shaped iced conductors (Figure 6), the variation in lift coefficient is more pronounced compared to the crescent-shaped case. The curve exhibits clear asymmetry, with a peak value slightly above 1.5 occurring near an angle of attack of approximately 90°. This sharp variation, accompanied by multiple local oscillation peaks, is attributed to the angular geometry of the sector shape. As the angle of attack changes, the flow separation point undergoes dynamic migration and sudden shifts along the surface. This unsteady behavior intensifies flow separation and reattachment, leading to strong shear layer oscillations and the formation of secondary vortex pairs. The resulting asymmetric wake vortex structures contribute to the observed local fluctuations in lift. In contrast, the drag coefficient is consistently higher than that of the crescent-shaped profile, reaching a maximum value of approximately 2.8 around a 100° angle of attack. This elevated drag is primarily due to the larger windward area and the wider, less streamlined trailing-edge wake of the sector-shaped geometry. Although the simulated peak values in the high-drag region were slightly lower than the experimental results, overall trends demonstrated strong agreement. The fitting accuracy is high across both low and high angles of attack, with the relative error remaining within acceptable limits.

**Figure 7:**
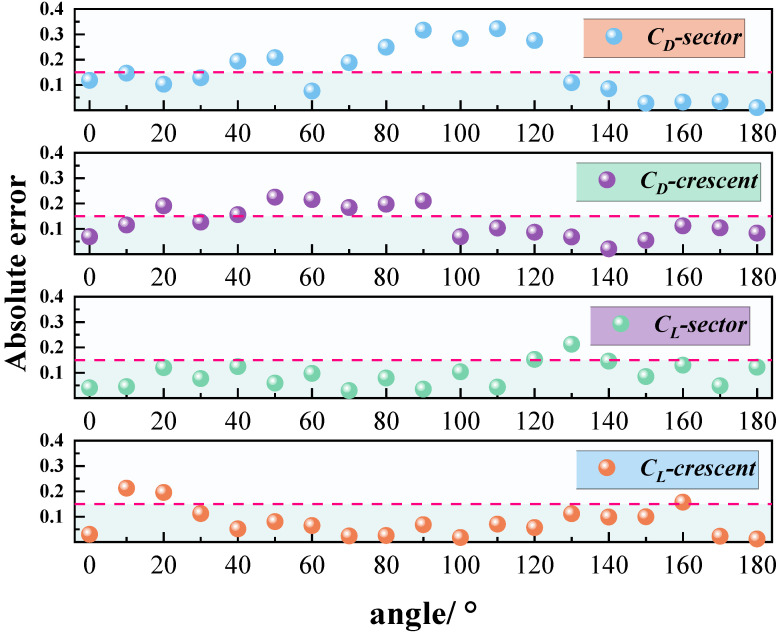
Absolute error of average lift coefficient and drag coefficient. (The dashed line in the figure represents an error value of 0.15, which serves to better compare the magnitude of the error values.)

It is worth noting that the lift coefficient curves include embedded local error magnification plots, highlighting the discrepancies between experimental and numerical results at critical angles of attack. For the crescent-shaped iced conductors, the lift error at an angle of approximately 110° reaches around 0.22. For the sector-shaped conductors, the drag error at 140° is approximately 0.18. These deviations are primarily attributed to the influence of turbulence intensity and surface roughness in the wind tunnel tests. While the numerical simulations capture the overall aerodynamic trends well, certain local discrepancies remain. Analysis of Figure 7 shows that, among the four typical conditions, the crescent-shaped conductors exhibit lower lift and drag errors compared to the sector-shaped ones. Notably, the lift coefficient errors for the crescent-shaped profile remain below 0.15 across most angles of attack, indicating strong consistency and simulation stability. In contrast, the sector-shaped conductors show larger drag coefficient errors, particularly in the 80–100° range, where the error exceeds 0.25 and exhibits significant fluctuations. This increased error is linked to the complex tail vortex structures of the sector shape, characterized by unsteady separation and asymmetric vortex interactions, which reduce simulation accuracy. Furthermore, the error curves remain relatively stable at low and high angles of attack, while the largest deviations occur in the mid-range angles. This region corresponds to the aerodynamic transition zone, where flow separation and reattachment are most dynamic. These findings suggest that future research should focus on improving the numerical accuracy and turbulence model adaptability in capturing separation behavior within this critical angle range.

Both crescent-shaped and sector-shaped iced conductors exhibit pronounced angle-of-attack sensitivity and nonlinear aerodynamic response characteristics. Among them, the sector-shaped conductors demonstrate a higher risk of aerodynamic instability and larger peak aerodynamic forces, which warrants particular attention in the design of wind-induced vibration mitigation measures. The numerical simulations successfully captured the overall experimental trends across the full range of angles of attack. Notably, the crescent-shaped profile shows better performance in reproducing peak aerodynamic forces and maintaining low error margins. This validates the reliability of the simulation approach and provides a solid data foundation for subsequent dynamic response and stability analyses.

**Figure 8:**
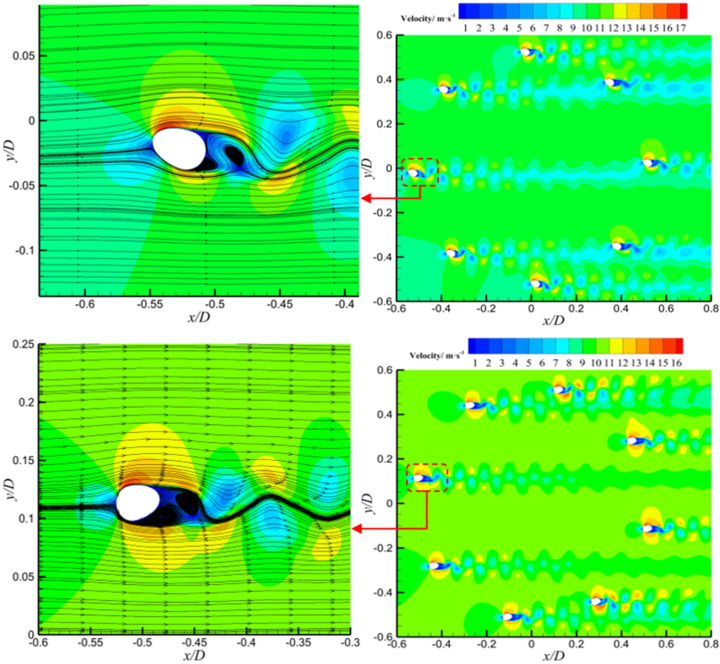
Velocity contour plots of 20° and 170° wind attack angles for crescent-shaped iced eight-bundled conductors.

Velocity contour plots were analyzed to investigate the bimodal behavior observed in the lift coefficient curve of crescent-shaped iced conductors. As shown in Figure 8, at an angle of attack of 20°, a prominent velocity gradient region is observed near the leading edge, indicating the onset of flow separation. The resulting shear layer extends downstream and develops into a periodic vortex structure. The wake region exhibits alternating bands of high and low velocity, characteristic of a classical Kármán vortex street. The lift spikes observed at this angle were likely caused by transient low-pressure regions forming on the leeward side during vortex shedding. As the vortex core is periodically shed from the conductor’s leeward surface, local asymmetry in the velocity field intensifies, resulting in a temporary increase in lift. At an angle of attack of 170°, the flow field exhibits strong asymmetry. The velocity distribution near the leading edge indicates reverse flow separation. In this case, the velocity gradient in the wake weakens, and the vortex structures appear more diffused. The second lift spike is believed to arise from unsteady separation induced by reverse flow conditions. When the incoming flow direction is nearly opposite to the conductor orientation, a transient high-velocity region forms near the trailing edge. This creates a strong velocity contrast with the upstream low-speed zone, thereby triggering abrupt lift fluctuations.

**Figure 9:**
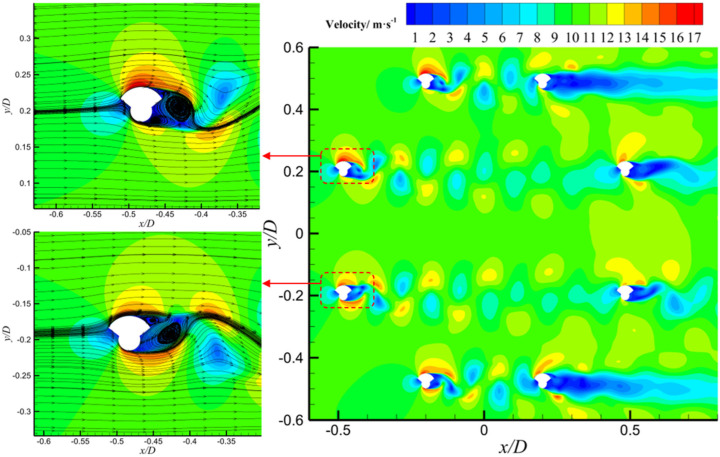
Velocity contour plot of 90° wind attack angles for sector-shaped iced eight-bundled conductors.

The single-peak behavior of the lift coefficient observed in sector-shaped iced conductors is closely associated with distinct flow separation features at an angle of attack of 90°. As shown in Figure 9, a pronounced velocity gradient region appears near the leading edge, indicating the onset of flow separation. The separated shear layer extends downstream, and the velocity distribution in the wake exhibits significant asymmetry. A broad low-velocity zone (velocity below 5 m/s) forms on the leeward side, while the windward side retains higher flow velocities in the range of 10–15 m/s. This asymmetric velocity field leads to a substantial pressure drop on the leeward side, resulting in a transient lift enhancement. At 90° angle of attack, the sharp angular geometry of the sector-shaped ice accretion causes strong interference with the incoming flow, resulting in a sudden displacement of the separation point from the leading edge to the midsection of the ice wing. This shift initiates shear layer oscillations and the formation of a localized recirculation zone with velocities below 3 m/s. The intensified velocity difference between windward and leeward sides further amplifies the pressure differential. Velocity contour plots of the wake region revealed intermittent formation of vortex cores on the leeward side, characterized by sharp velocity gradients. The asymmetric evolution of these vortex cores contributes to increased amplitudes in instantaneous lift fluctuations. From the above analysis, it can be concluded that the lift spike at 90° for sector-shaped iced conductors is primarily driven by geometry-induced separation instability and the resulting unsteady, asymmetric vortex structure in the wake.

The primary validation of the CFD simulations, as presented in Section 3.1, focused on comparing the computed static aerodynamic coefficients—lift, drag, and moment—with corresponding wind tunnel measurements. The results show that good agreement was achieved between the numerical and experimental results for integrated aerodynamic forces, confirming the reliability of the CFD approach for evaluating the key aerodynamic parameters relevant to galloping analysis. Direct velocity field measurements (e.g., using Particle Image Velocimetry or hot-wire anemometry) were not conducted in this study due to experimental setup constraints and the primary focus on force-based validation. Nonetheless, we acknowledge the importance of detailed flow field comparisons for comprehensively verifying simulated flow structures. The recent literature demonstrates the value of advanced flow visualization and measurement techniques in capturing local flow features with high spatial and temporal resolution [34,35]. Incorporating such methods into future studies will be essential for further validating the detailed dynamics of unsteady flow phenomena predicted by numerical simulations.

### 4.2. Dynamic Aerodynamic Response

The dynamic aerodynamic characteristics of crescent-shaped and sector-shaped iced eight-bundled conductors over the full angle-of-attack range (0–180°) were investigated through a combined analysis using fourth-order Fourier fitting and harmonic energy spectrum analysis. This approach reveals the distinct regulatory effects of ice geometry on flow separation and vortex shedding behavior. By quantifying the fitting accuracy, harmonic energy distribution, and its relationship with transient flow structures, a multiscale analytical framework is established. This framework captures the nonlinear aerodynamic responses, frequency-domain energy transfer mechanisms, and flow field evolution associated with different ice accretion profiles. The findings offer a new perspective for the theoretical modeling of galloping in iced conductors and contribute to a deeper understanding of the aerodynamic instability mechanisms involved.

**Figure 10:**
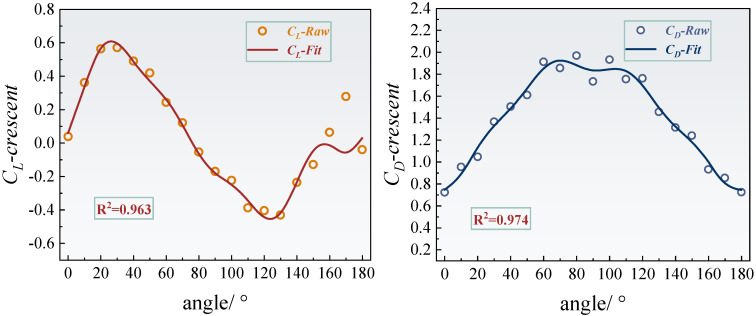
Fourth-order Fourier fitting curves and goodness-of-fit of lift and drag coefficients for crescent-shaped iced eight-bundled conductors.

**Figure 11:**
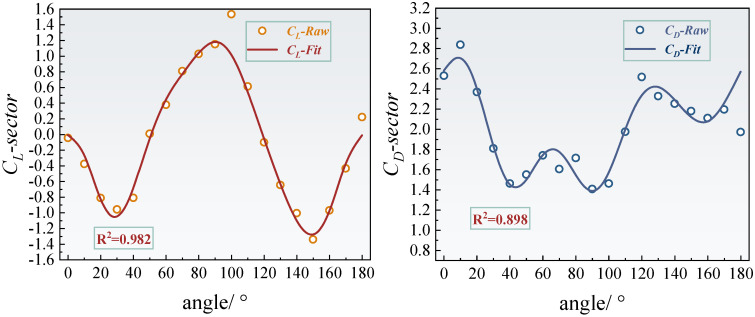
Fourth-order Fourier fitting curves and goodness-of-fit of lift and drag coefficients for sector-shaped iced eight-bundled conductors.

**Figure 12:**
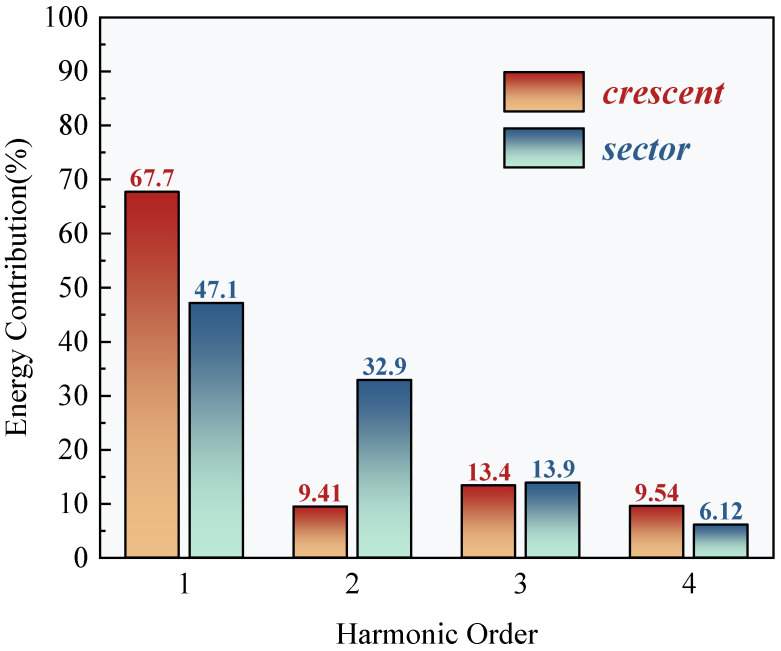
Fourth-order harmonic energy.

As shown in Figure 10 and Figure 11, the fourth-order Fourier fitting results demonstrate that the lift and drag coefficients of the crescent-shaped iced conductors achieve goodness-of-fit values of 0.963 and 0.974, respectively. Their dynamic responses exhibit typical quasi-periodic characteristics. Specifically, the lift coefficient reaches a local minimum of 0.24 at an angle of attack of 60°, and a pronounced negative peak of −0.44 at 120°, reflecting nonlinear fluctuations closely related to asymmetric flow separation near the leading edge of the crescent-shaped ice. Figure 12 further reveals that the dynamic response of the crescent-shaped conductors is predominantly governed by the first-order harmonic, which accounts for 67.7% of the total spectral energy. The contributions from the second- and third-order harmonics are significantly smaller, at 9.41% and 13.4%, respectively. This strong dominance of the first harmonic suggests a relatively singular and stable vortex shedding frequency, indicative of a quasi-steady Kármán vortex street. Such concentrated spectral energy at a single dominant frequency results in more predictable and stable aerodynamic forcing. Consequently, the galloping tendency is reduced and can often be mitigated effectively using conventional damping strategies designed for narrow-band excitation. In contrast, sector-shaped iced conductors exhibited a slightly higher goodness-of-fit for the lift coefficient (0.982), but significantly lower for the drag coefficient (0.898). This discrepancy suggests increased flow instability in certain regions, likely caused by secondary separation or asymmetric vortex merging phenomena. The harmonic energy spectrum supports this interpretation: the second-order harmonic contribution rises markedly to 32.9%, and the third-order component reaches 13.9%. This broadband energy distribution is directly linked to the dynamic migration of the separation point, a behavior induced by the prismatic geometry of the sector-shaped ice. In such cases, the separation point can abruptly shift from the leading to the trailing edge, generating shear layer oscillations and secondary vortex pairs. These interactions triggered multi-frequency aerodynamic excitations. The dispersion of energy across multiple harmonics indicates a more complex and unstable aerodynamic forcing environment. This complexity poses greater challenges for galloping prediction and control, necessitating broader or multi-band mitigation strategies to effectively disrupt energy transfer pathways and suppress aerodynamic instability.

Harmonic energy spectrum analysis quantitatively assessed how ice accretion geometry modulates energy distribution in the frequency domain. The first-order harmonic energy contribution for the crescent-shaped iced conductors is significantly higher than that of the sector-shaped case. In contrast, the second-order harmonic energy in the sector-shaped configuration is approximately 3.5 times greater than that of the crescent-shaped profile. The physical origin of these differences lies in the distinct flow field evolution mechanisms associated with each ice shape. The streamlined leading edge of the crescent-shaped conductors promotes stabilization of the separation point near the trailing edge. This leads to the formation of a dominant single vortex shedding mode, where energy input is primarily concentrated at a single frequency, corresponding to the first harmonic. In contrast, the angular geometry of the sector-shaped ice induces abrupt shifts in the separation point with varying angles of attack. The resulting asymmetrical interactions between unsteady shear layer oscillations and secondary vortex pair formations stimulate a stronger second-order harmonic response. Moreover, the dynamic merging and splitting of vortex cores further excite third-order harmonic components, contributing to a broadband energy distribution. This mechanism introduces complex, multi-frequency aerodynamic excitation, characteristic of a more unstable and less predictable flow regime.

The synergistic analysis reveals a strong coupling between the frequency-domain characteristics of aerodynamic forces and underlying flow field instability mechanisms, which directly influences galloping susceptibility. For crescent-shaped icing, the dominance of a single frequency component indicates a more coherent and stable aerodynamic excitation. This concentrated energy input can typically be mitigated effectively using conventional damping strategies, which are designed to dissipate energy within a narrow frequency range, thereby improving galloping stability. In contrast, the broadband energy distribution observed in sector-shaped icing—marked by significant contributions from multiple harmonic components—indicates a more complex and potentially unstable aerodynamic response. This condition necessitates the use of multiband tuning or broadband damping strategies to effectively disrupt energy transfer across various frequencies. The presence of multiple excitation modes complicates the prediction and effective mitigation of galloping behavior. Furthermore, the substantial increase in second-order harmonic energy in sector-shaped conductors suggests a possible correlation with the occurrence of negative aerodynamic damping, as described by the Den Hartog criterion. However, a precise quantification of this relationship requires further investigation in conjunction with dynamic stability analysis.

### 4.3. Stability and Flow Field Mechanisms

**Figure 13:**
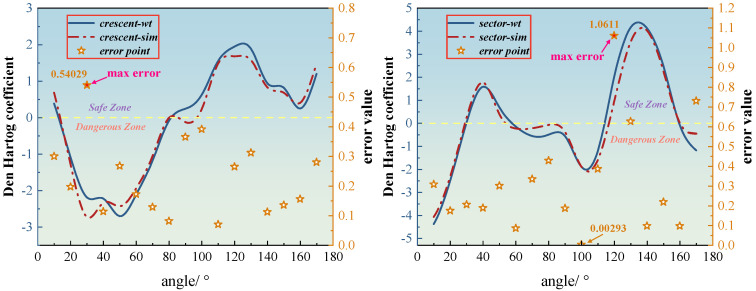
Den Hartog coefficients and errors for crescent-shaped and sector-shaped iced eight-bundled conductors. (The dashed line is the standard line for the Den Hartog criterion.)

To further assess the aerodynamic stability of iced conductors across different angles of attack, the distribution of the Den Hartog coefficient was calculated based on both wind tunnel test results and numerical simulations. A comparative analysis was then conducted using the experimental data. The Den Hartog coefficients for crescent-shaped and sector-shaped iced conductors over the 0–180° angle-of-attack range are presented in Figure 13. In the figure, solid lines represent the wind tunnel measurements, dashed lines denote the simulation results, and five-pointed stars indicate the error between the two datasets. To facilitate interpretation, a horizontal dashed line is drawn at *H* = 0 to demarcate the stability threshold. Regions where the Den Hartog coefficient is negative are labeled as the “Dangerous Zone” (indicating negative aerodynamic damping and potential galloping risk), while regions with positive coefficients are labeled as the “Safe Zone” (indicating positive damping and aerodynamic stability). This visual classification helps identify critical working conditions where galloping is more likely to occur.

For the crescent-shaped iced conductors, the Den Hartog coefficient remains negative between angles of attack of 20° and 70°, reaching a minimum near −3.2 at approximately 40°, indicating a high risk of aerodynamic self-excited galloping within this range. The coefficient becomes positive and stabilizes at higher values from 80° to 130°, suggesting a lower susceptibility to wind-induced vibration in this region. Overall, the experimental and numerical curves exhibit strong agreement, with the maximum discrepancy at critical points remaining within 0.6. Specifically, the largest error of about 0.54 occurs around 30°, highlighting the difficulty in accurately capturing the transition from negative to positive damping. The lowest error, approximately 0.07 at 110°, demonstrates that the SST *k*-*ω* turbulence model is highly accurate for simulating near-wall flow conditions at lower angles of attack. In contrast, sector-shaped iced conductors exhibit a broader “Dangerous Zone,” maintaining negative Den Hartog coefficients across 10 angle conditions, with the lowest value approaching −4.5. This indicates a notably higher and more persistent risk of aerodynamic instability. Although the maximum positive coefficient in the “Safe Zone” reaches approximately 4.0, the corresponding numerical simulation errors are significantly higher, peaking at around 1.06. Such substantial deviations suggest that turbulent separation and nonlinear wake dynamics pose significant challenges for accurate numerical prediction. This elevated error likely arises from inherent limitations in the computational methodology when applied to complex geometries and flow conditions. Firstly, the highly unsteady flow around the sharp-edged sector geometry involves dynamic shifts of the separation point and asymmetric vortex shedding, phenomena not fully captured by the steady RANS equations, even with advanced turbulence models like SST *k*-*ω.* By design, RANS models smooth out transient flow fluctuations, thus limiting their accuracy for flows with pronounced separation and reattachment. Secondly, despite careful mesh generation efforts, mesh resolution limitations—particularly in regions of dynamic separation and highly turbulent wakes—may have prevented adequate resolution of fine-scale turbulent structures. Capturing these intricate, unsteady flow phenomena with greater fidelity would necessitate employing computationally more demanding methods, such as Large Eddy Simulation (LES) or Direct Numerical Simulation (DNS). Although beyond the scope of the current study, these methods represent important avenues for future research aimed at enhancing prediction accuracy in such complex aerodynamic regimes.

Based on the above analysis, the crescent-shaped iced conductors exhibit better aerodynamic stability than their sector-shaped counterparts. They are characterized by narrower instability intervals and smaller simulation errors, making them more suitable for direct application in wind-induced vibration prediction and galloping prevention design. In contrast, sector-shaped iced conductors show a higher likelihood of galloping across a broader range of operating conditions. This indicates that traditional galloping mitigation strategies may require modification or innovation to address the more complex aerodynamic behavior associated with this geometry.

## 5. Conclusions

This study conducted a comprehensive investigation of the aerodynamic characteristics and instability mechanisms of crescent-shaped and sector-shaped iced eight-bundled conductors through a synergistic approach that integrated wind tunnel testing with high-fidelity CFD simulations. The strong agreement between simulation results and experimental measurements confirms the reliability of the numerical method and provides a robust foundation for subsequent analyses.

This study employed a synergistic approach combining wind tunnel experiments and CFD simulations to investigate the aerodynamic instability mechanisms of iced bundled conductors. A key contribution of this work lies in the detailed frequency-domain analysis, which offers novel insights into the distinct dynamic behaviors associated with different ice accretion geometries. For crescent-shaped iced conductors, the streamlined leading edge facilitates stable flow separation, resulting in a dynamic response dominated by first-order harmonics and a single, well-defined vortex shedding frequency. This behavior aligns with established aerodynamic theory and further substantiates the lower galloping risk typically associated with crescent-shaped ice under real conditions. In contrast, the sharp prismatic geometry of sector-shaped iced conductors induces abrupt and dynamic shifts in the separation point. This leads to a broadband aerodynamic excitation spectrum, characterized by significant energy contributions from both second- and third-order harmonics. This broadband behavior, quantitatively analyzed in this study for the first time, provides a clear explanation for the increased aerodynamic instability observed in field conditions. The findings offer a deeper understanding of the severe galloping incidents frequently associated with sharp-edged ice shapes and underscore the need for more robust mitigation strategies in such scenarios.

Den Hartog coefficient analysis reveals substantial differences in aerodynamic stability between the two ice accretion geometries. For crescent-shaped iced conductors, negative damping—indicative of instability—is observed under six angle-of-attack conditions, primarily between 20° and 70°, with a minimum coefficient of approximately −3.2 occurring near 40°. In contrast, sector-shaped iced conductors exhibit a significantly broader “Dangerous Zone”, with negative damping present under ten angle-of-attack conditions. This reflects an increase of four unstable cases compared to the crescent shape, representing a more than 60% expansion in the range of instability. Moreover, the minimum Den Hartog coefficient for the sector-shaped conductors reaches nearly −4.5, indicating a roughly 40% increase in negative damping intensity relative to the crescent-shaped case. The agreement between numerical predictions and wind tunnel measurements of the Den Hartog coefficients exceeds 90% across most angles of attack, demonstrating the reliability of the simulation approach for evaluating galloping susceptibility.

This study employed the RANS equations with the SST *k*-*ω* turbulence model to perform CFD simulations. This approach offers a favorable balance between computational efficiency and robust performance in capturing complex aerodynamic flows and separation phenomena. A detailed validation against wind tunnel data provided a comprehensive assessment of the model’s applicability. The SST *k*-*ω* model demonstrated high accuracy in predicting lift coefficients, particularly for crescent-shaped iced conductors, with errors remaining below 5%. This confirms the model’s capability to reliably capture vertical aerodynamic forces, which are critical for galloping risk assessment in actual transmission lines. However, drag coefficient predictions exhibited slightly larger discrepancies, with maximum errors reaching approximately 0.3. These deviations are primarily attributed to the model’s inherent limitations in accurately resolving highly unsteady and asymmetric vortex merging in the wake region. This identification of specific error sources offers valuable diagnostic insight into the challenges of CFD modeling for geometrically complex, ice-covered conductors. Overall, the strong agreement between numerical and experimental results confirms that the selected modeling approach is a reliable and practical tool for predicting key aerodynamic characteristics. It effectively supports the practical analysis and mitigation of galloping in real transmission line applications.

The synergistic analysis of Fourier harmonic energy and CFD-based transient flow fields suggests that the second-order harmonic energy contribution can serve as a frequency-domain early warning indicator for galloping risk. This finding provides a theoretical basis for optimizing the frequency bandwidth of galloping mitigation devices in ultra-high voltage transmission lines.

These findings provide critical guidance for the design, risk assessment, and development of targeted anti-galloping measures for transmission lines. In particular, they underscore the need for advanced mitigation strategies to address the aerodynamic complexities associated with ice accretion geometries such as the sector shape.

## Figures and Tables

**Table 1:** Comparison of mesh density.

Ice Type	Mesh Density (in Millions)	Lift Coefficients of Conductors 1	Parameter 1 Errors	Strouhal Number	Parameter 2 Errors
crescent	3	0.446	4.8%	0.246	0.8%
crescent	4	0.423	0.5%	0.247	0.2%
crescent	5	0.425	comparison	0.248	comparison
sector	3	−0.082	9.9%	0.152	5.5%
sector	4	−0.089	2.3%	0.148	2.4%
sector	5	−0.091	comparison	0.144	comparison

## Data Availability

Data available on request from the authors.
